# Clinical Value of Combined Determination of Serum B7-H4 with Carcinoembryonic Antigen, Osteopontin, or Tissue Polypeptide-Specific Antigen for the Diagnosis of Colorectal Cancer

**DOI:** 10.1155/2018/4310790

**Published:** 2018-09-27

**Authors:** Peng Wang, Chun Li, Fan Zhang, Xuzhe Ma, Xiaodong Gai

**Affiliations:** ^1^Department of Pathology, Medical School of Beihua University, Jilin, China; ^2^Key Laboratory of Molecular Medicine, Jilin Province Education Department, Beihua University, Jilin, China

## Abstract

**Aim:**

B7-H4 is member of the B7 family that negatively regulates the immune response, which are associated with tumor development and prognosis. The present study is aimed at examining serum B7-H4 expression and exploring its contribution to diagnosis in patients with colorectal cancer.

**Methods:**

We determined serum expressions of B7-H4, carcinoembryonic antigen (CEA), osteopontin (OPN), and tissue polypeptide-specific antigen (TPS) in 59 patients with colorectal cancer and 29 healthy volunteers and analyzed the diagnostic value of B7-H4 combined with CEA, OPN, or TPS detection for colorectal cancer. B7-H4, OPN, and TPS serum expressions were measured by enzyme-linked immunosorbent assay, and CEA was measured by electrochemical luminescence detection.

**Results:**

Serum B7-H4 levels were significantly higher in colorectal cancer patients compared with paired normal controls (*P* = 0.001). B7-H4 serum level was positively correlated with infiltration depth, tumor masses, and lymph node metastasis (*P* = 0.004, *P* = 0.016, and *P* = 0.0052, respectively). We also detected serum expression of B7-H4 before and after radical resection and showed that B7-H4 levels decreased significantly during the first week postoperation (*P* = 0.0064). We used receiver operating characteristic (ROC) curve analysis to indicate the potential diagnostic values of these markers. The areas under the ROC curves (AUC) for B7-H4, OPN, TPS, and CEA were 0.867, 0.805, 0.812, and 0.833, respectively. The optimal sensitivity and specificity of B7-H4 for discriminating between colon cancer patients and healthy controls were 88.2% and 86.7%, respectively, using a cut-off of value of 78.89 ng/mL. However, combined ROC analysis using B7-H4 and CEA revealed an AUC of 0.929, with a sensitivity of 98.9% and a specificity of 80.4% for discriminating colon cancer patients from healthy controls.

**Conclusions:**

B7-H4 was highly expressed in the serum in colorectal cancer patients. Detection of B7-H4 plus CEA showed significantly increased sensitivity and specificity for discriminating between colorectal cancer patients and healthy controls compared to individual detection of these markers. Combined detection of serum B7-H4 and CEA may thus have the potential to become a new laboratory method for the early clinical diagnosis and prognostic evaluation of colorectal cancer.

## 1. Introduction

Colorectal cancer (CRC) is one of the most frequent cancers diagnosed in high-income countries and most middle-income countries [1], accounting for nearly 8.5% of total cancer-related death annually [2, 3]. Moreover, many patients with CRC are not diagnosed until the disease is in its advanced stages and is no longer treatable. Extensive research into the factors related to CRC suggests a crucial role of early diagnosis, though several studies have failed to prove a causal relationship [1]. Tumor initiation and development have been shown to be complex processes involving multiple stages and regulation of multiple genes. Although CRC develops as a consequence of local tissue changes [4, 5], methods of predicting cancer risk before the appearance of morphological changes in the colon remain unclear.

Successful detection of CRC in the early stages is crucial for providing effective therapies for this aggressive and malignant disease, and screening for sensitive and specific tissue or body-fluid biomarkers that may provide an accurate early diagnosis of CRC has been an important task for both clinicians and cancer biologists in recent decades. Although carcinoembryonic antigen (CEA) is currently the most commonly used serum tumor marker for CRC [6–9], it is not recommended as a screening or diagnostic tool for this neoplasm, especially in the early stages. B7-H4 is a B7 family molecule and a highly evolutionarily conserved transmembrane protein that shares approximately 25% amino acid homology in the extracellular portion with other B7 family members [10]. B7-H4 ligation of T cells causes cell cycle arrest and has a profound inhibitory effect on cell growth, cytokine secretion, and the development of cytotoxicity [11]. Administration of B7-H4 Ig to mice impaired antigen-specific T-cell responses, whereas blockade of endogenous B7-H4 by specific monoclonal antibody promoted T-cell responses [12].

B7-H4 can regulate the T-cell immune response by inhibiting T-cell proliferation, cytokine production, and the cell cycle [13] and may thus participate in the negative regulation of cell-mediated immunity in normal peripheral tissues [12]. In contrast, B7-H4 has been shown to be overexpressed in some cancers, such as ovarian cancer, lung cancer, and breast cancer [14–16]. We previously demonstrated that B7-H4 had a strong prognostic significance and promoted tumor tolerance and might contribute to Treg development in the CRC tolerogenic milieu [17]. Higher serum B7-H4 levels have also been reported in CRC patients and were closely correlated with many important clinicopathological parameters, further supporting its potential value for the early diagnosis and prediction of disease prognosis.

In addition to B7-H4, numerous tumor markers have been found and used in clinical practice [18]. Tumor markers may reflect the presence and growth of the tumor, thus providing information on the nature of the tumor and potentially aiding its early diagnosis. However, the expression pattern and diagnostic value of B7-H4 protein have not yet been investigated. This study therefore investigated the serum expression levels of B7-H4 in patients with CRC. We also explored the diagnostic sensitivity and accuracy of B7-H4 in combination with serum osteopontin (OPN) [19], tissue polypeptide-specific antigen (TPS) [20], and CEA [21] for the early detection of CRC.

## 2. Materials and Methods

### 2.1. Patients and Serum Samples

This study was approved by the Research Ethics Committee of the General Hospital of Jilin Chemical Industry, China. Written informed consent was obtained from all the patients according to the committee's regulations. All samples were handled and anonymized according to the ethical and legal standards. Fifty-nine paraffin-embedded CRC samples and matched serum samples were provided by the General Hospital of Jilin Chemical Industry for patients with complete histopathology and follow-up information from 2013 to 2014. None of the patients received preoperative chemotherapy or radiotherapy. The clinicopathological characteristics of the patients are detailed in Table 1.

Twenty-four healthy age- and sex-matched controls were recruited from among people who came for general health examinations. Blood samples were kept at room temperature for 22 h, and serum was obtained by centrifugation at 3000×g at 4°C for 15 min. The serum was removed immediately and frozen on dry ice at −80°C until use.

### 2.2. Measurement of Serum B7-H4, OPN, and TPS

B7-H4, OPN, and TPS serum levels were measured by enzyme-linked immunosorbent assay (ELISA) methods, as described previously [22]. B7-H4 levels were also determined using an assay kit (Rapidbio, Hayward, CA, USA). The sensitivities of the B7-H4 immunoassays were 0.156 ng/mL. Briefly, 100 *μ*L of serum was placed in each well of the ELISA plate and incubated for 45 min at 37°C. The plates were washed four times with buffer and incubated with 50 *μ*L of detection antibody (0.2 *μ*g/mL) at 37°C for 1 h. After five washes, the plates were incubated with 100 *μ*L horseradish peroxidase solution (1 : 5000) for 30 min at 37°C and then reacted with the enzyme substrate p-nitrophenyl phosphate (Cat N1891, Sigma–Aldrich, WI, USA) for 30 min at room temperature. The absorbance was then read at 450 nm using a microplate ELISA reader (Multiskan MK3, Thermo Scientific, MA, USA). A corresponding standard curve was generated using the provided standards and was used to calculate the quantities of B7-H4, OPN, and TPS in each serum sample.

### 2.3. Electrochemical Luminescence Detection of Serum CEA

Levels of CEA in the serum samples were measured using a two-site microtiter plate-based immunoassay with electrochemical luminescence detection (Cobas601 Kit, Roche, Germany) according to the manufacturer's instructions. At least three independent experiments were conducted for each case. The accuracy of the results for the quality control samples and sensitivity met the experimental requirements.

### 2.4. Statistical Analysis

Data are expressed as mean ± standard deviation. Comparisons among multiple groups and between two groups were conducted using analysis of variance with Tukey's post hoc test and Student's *t*-test, respectively. Variables following a nonnormal distribution were logarithmically transformed (natural logarithm) before use in parametric analyses. Pearson correlation and *χ*^2^ test or Fisher's exact test were used for comparison and estimation of correlations between B7-H4, OPN, TPS, and CEA serum expressions and clinicopathological tumor parameters as infiltration, metastasized, lymph node metastasis, or differentiation. Specificity and sensitivity of serum B7-H4, OPN, TPS, and CEA expression levels for CRC patients with control of follow-up were evaluated with receiver operating characteristic (ROC) curve analysis. Diagnostic accuracy of biomarkers was also determined by obtaining the largest possible area under the curve (AUC) in ROC analysis. Bonferroni data were analyzed using SPSS v.23.0 software. For all comparisons, *P* < 0.05 was considered statistically significant. Graphs were plotted using GraphPad Prism v.6.0 software (GraphPad Inc., La Jolla, CA, USA).

## 3. Results

### 3.1. Comparison of B7-H4, OPN, TPS, and CEA Levels between CRC Patients and Controls

We analyzed B7-H4, OPN, TPS, and CEA protein levels in 59 CRC and 29 corresponding normal serum samples. The mean B7-H4 serum level in CRC patients (95.78 ± 13.42 ng/mL) was significantly higher than that in healthy controls (63.20 ± 15.69 ng/mL) (*P* = 0.001). The mean OPN, TPS, and CEA serum expressions were also significantly higher in CRC samples compared with controls (OPN: 116.14 ± 36.31 vs. 78.69 ± 16.45 ng/mL, *P* = 0.032; TPS: 98.77 ± 27.62 vs. 76.40 ± 15.04 U/L, *P* = 0.018; CEA: 77.17 ± 81.09 vs. 1.95 ± 1.04 ng/mL, *P* = 0.008). Summarized data for all markers are shown in Table 2. There was no significant correlation between B7-H4, OPN, TPS, or CEA expression and age or gender (Table 3).

### 3.2. Diagnostic Value by Receiver Operating Characteristic Curve Analysis

**Figure 1:**
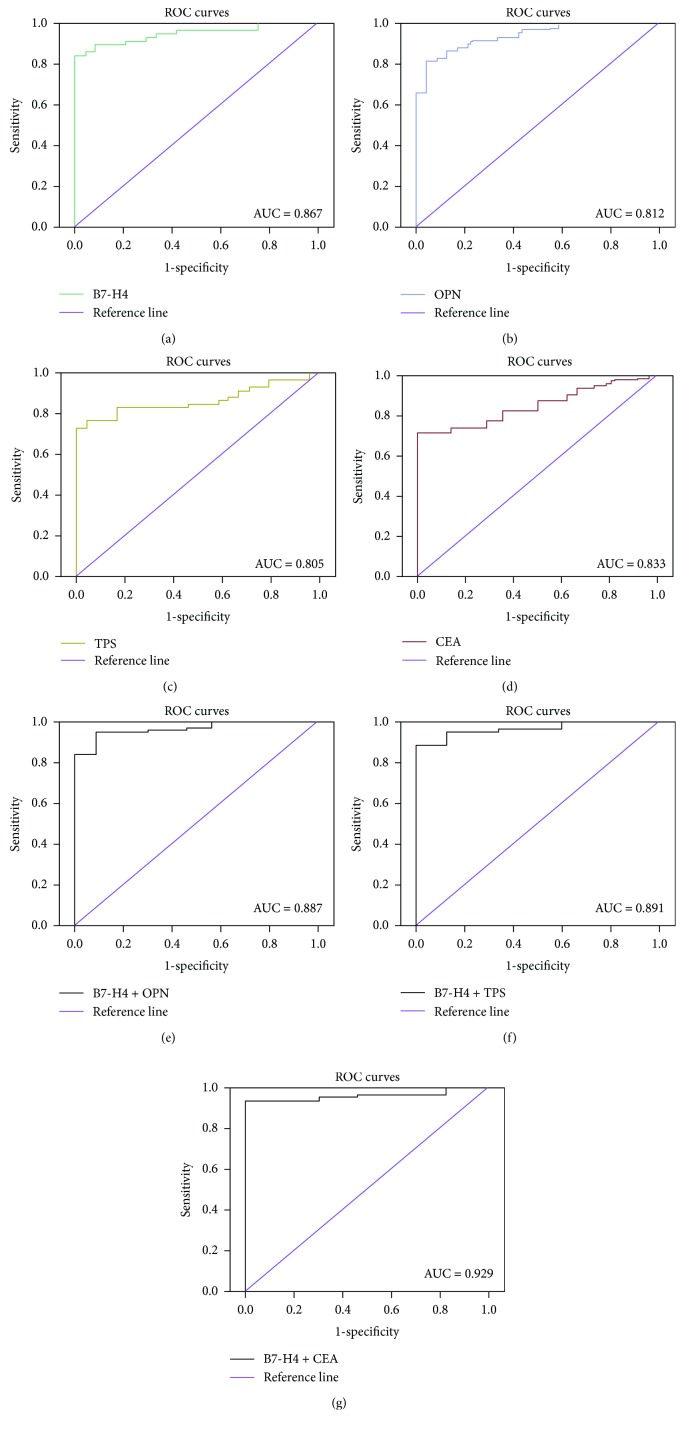
ROC curves of B7-H4/OPN/TPS/CEA of CRC patients. Receiver operating characteristic (ROC) curve analysis using four biomarkers to differentiate patients. Diagnostic accuracy of biomarkers was determined by obtaining the largest possible area under the curve (AUC) in ROC analysis. (a) B7-H4, (b) OPN, (c) TPS, (d) CEA, (e) B7-H4 + OPN, (f) B7-H4 + TPS, and (g) B7-H4 + CEA.

**Figure 2:**
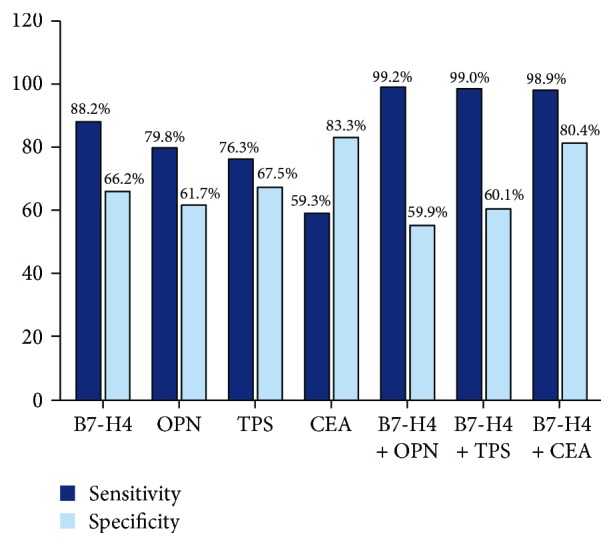
The diagnosis value of serum B7-H4/OPN/TPS/CEA in CRC patients. Combined analysis using B7-H4 and CEA revealed sensitivity of 98.9% and specificity of 80.4% for discriminating CRC patients from healthy controls.

The above results and previous reports [17–19, 22, 23] indicated that serum expressions of B7-H4, OPN, TPS, and CEA were markedly higher in tumor patients than in healthy controls. We investigated the potential diagnostic roles of these markers in CRC by receiver operating characteristic (ROC) curve analysis and the area under curve (AUC). The cut-off values based on the ROC analysis were 78.89 ng/mL for B7-H4, 95.06 ng/mL for OPN, 91.44 U/L for TPS, and 3.4 ng/L for CEA. The ROC curve demonstrated optimal sensitivity (0.882) of B7-H4 serum levels for distinguishing between CRC patients and healthy controls at a threshold of 78.89 ng/mL, with an AUC of 0.867, while CEA levels showed the highest specificity (0.833) (Figure 1). Combined ROC curve analysis using B7-H4 and CEA revealed an AUC of 0.929 with a sensitivity of 98.9% and a specificity of 80.4% for discriminating CRC patients from healthy controls. (Figure 1 and Table 4). While the AUC of combinational B7-H4 and OPN is 0.887 with sensitivity of 99.2% and specificity of 55.9%, ROC curve of combined B7-H4 and TPS revealed an AUC of 0.891 with a sensitivity of 99.0% and a specificity of 60.1%. Among these biomarkers, the best accuracy (Youden's index) of diagnosis for CRC was B7-H4 and CEA (0.793) (Table 4). The results indicated that combined detection using B7-H4 and CEA significantly improved the sensitivity and specificity for diagnosing CRC (Figure 2).

### 3.3. Correlation between Serum Expressions of B7-H4, OPN, TPS, and CEA and Clinicopathological Parameters of CRC

The correlations between serum B7-H4, OPN, TPS, and CEA protein levels and clinicopathologic features are summarized in Table 3. There was no significant correlation between TPS (*P* = 0.962) or CEA (*P* = 0.365) expression and tumor infiltration. However, high serum expressions of B7-H4 (*P* = 0.0001) and OPN (*P* = 0.040) were significantly correlated with tumor infiltration. B7-H4 (*P* = 0.016) and TPS (*P* = 0.027) levels were also significantly correlated with tumor masses, while OPN (*P* = 0.567) and CEA (*P* = 0.253) levels were not related to CRC tumor masses (Table 3). B7-H4 serum expression was significantly higher in CRC patients with more lymph node metastases (≥4) (110.70 ± 16.52 ng/mL) compared with patients with fewer lymph node metastases (91.945 ± 19.43 ng/mL) (*P* = 0.0052), suggesting that B7-H4 was related to tumor progression and metastasis in CRC. We also evaluated the associations of serum B7-H4, OPN, TPS, and CEA levels with distant metastases in patients with CRC. The mean serum level of B7-H4 was significantly higher in patients with advanced tumor stage compared in those with early tumor stage (*P* = 0.017), and Mann–Whitney *U* analysis also showed that B7-H4, OPN, and TPS serum expressions were significantly related to distant metastasis (*P* = 0.0041, *P* = 0.015, and *P* = 0.021, respectively). However, there was no relationship between CEA levels (*P* = 0.069) and distant metastasis (Table 3).

### 3.4. Assessment of Preoperative and Postoperative Serum Markers

**Figure 3:**
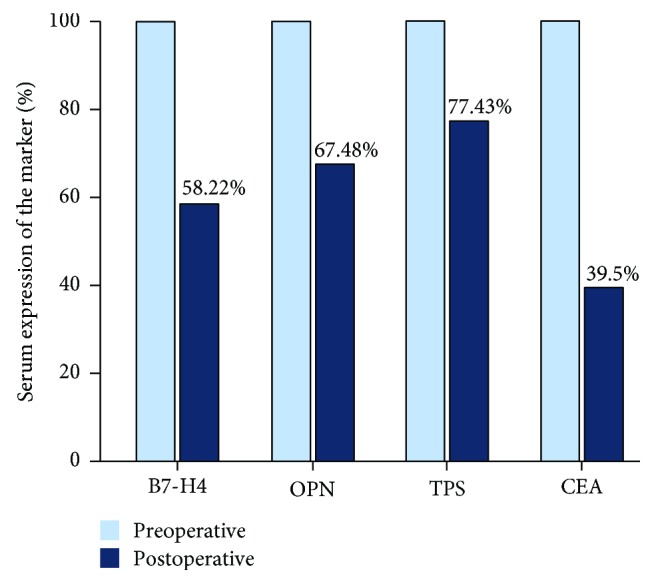
The comparison of serum concentrations of B7-H4/OPN/TPS/CEA between preoperative and postoperative groups in CRC patients.

All 59 patients were evaluated preoperatively before radical resection for CRC. Twenty-one patients also underwent peripheral lymph node dissection. No patients experienced surgical or perioperative mortality, and there were no perioperative complications including hemorrhage and intestinal fistula. Serum expressions of B7-H4, OPN, TPS, and CEA detected again on the 7th postoperative day had all decreased significantly compared with preoperative levels (*P* = 0.0064, *P* = 0.041, *P* = 0.016, and *P* = 0.012, respectively) (Table 5, Figure 3) and were similar to the levels in the healthy control group (*P* < 0.05). These results confirmed the relationships between these biomarkers and clinicopathologic features in CRC.

## 4. Discussion

China has experienced a two- to fourfold increase in the incidence of CRC during the past few decades [18, 19]. Although changes in dietary habits and lifestyle are believed to largely account for this increase, the interaction between environmental and genetic factors might play a pivotal role in colorectal carcinogenesis in Asian populations. Although early diagnosis and treatment have greatly improved the prognosis of this aggressive neoplasm [24, 25], the almost complete lack of symptoms in the early stages and the absence of highly sensitive, effective, and economical methods of screening make it difficult to detect CRC before dissemination of the tumor. It would therefore be useful to identify individual or combined serum tumor markers that could aid in the early detection of CRC.

B7-H4 is a novel member of the inhibitory B7 family and is regarded as a negative regulator of the T-cell-mediated immune response and a potential diagnostic marker for CRC [17]. Previous studies demonstrated that B7-H4 was highly expressed in many different types of human cancers and was mostly associated with poor clinical outcomes [26]. B7-H4 was overexpressed in most CRC tumor tissues and was associated with infiltration depth and lymph node metastasis, which are associated with a poorer clinical outcome in patients with CRC [17, 27]. However, although serum B7-H4 levels have been considered to have diagnostic value in several human cancers, information on its use for the early diagnosis in CRC is currently lacking.

In the current study, we measured B7-H4 levels in CRC serum samples and showed that mean serum expression was significantly higher in CRC patients compared with healthy controls. These results were consistent with previous studies that reported increased B7-H4 expression in blood samples from patients with ovarian cancer, gastric cancer, and renal cell carcinoma [28, 29]. Serum B7-H4 might thus serve as a potential biomarker of CRC. We further analyzed the correlations between serum B7-H4 and clinicopathologic features of CRC and showed that B7-H4 overexpression was closely associated with tumor size, lymph node metastasis, and tumor infiltration but not with gender or age. Using a combination of different biomarkers has been reported to be a useful strategy [30–33] by increasing the sensitivity and specificity compared with the individual biomarkers alone. Although serum expressions of other tumor markers such as OPN, TPS, and CEA differed between CRC patients and healthy controls, they were not closely related to the clinicopathologic parameters of CRC, thus emphasizing the importance of B7-H4 in the development and progression of CRC. Moreover, higher serum expression of B7-H4 in CRC patients was also correlated with distant metastases, indicating that serum B7-H4 may be a potential diagnostic marker for distant metastases in CRC patients.

We further evaluated serum B7-H4 levels before and 7 days after radical resection of CRC and showed that B7-H4 levels decreased significantly during the first postoperative week, then recovered to normal. Given the positive correlation between B7-H4 protein expression in CRC tumor tissues [17], these results suggest that assessment of serum expression of B7-H4 after surgery might provide a tool for assessing the success and curative effect of surgery in CRC patients.

We also determined if the combined detection of B7-H4 with OPN, TPS, or CEA could increase the specificity and sensitivity of B7-H4 for CRC diagnosis. B7-H4 alone exhibited high sensitivity and low specificity, while CEA exhibited the opposite properties. OPN and TPS did not have high sensitivity or specificity for CRC diagnosis. We performed ROC analysis using the two markers combined and showed that the combination provided relatively high sensitivity and specificity for discriminating between CRC patients and healthy controls. No reliable independent biomarker has yet been established for CRC screening, and the current results suggest that the combination of B7-H4 and CEA serum assays may offer a useful tool for the detection of CRC.

In summary, elevated serum expression of B7-H4 may play a critical role in the development and progression of CRC. Detection of B7-H4 might thus serve as a clinical predictor of the diagnosis or outcomes in patients with CRC. The combined detection of B7-H4 and CEA significantly increased the sensitivity and specificity of CRC diagnosis compared to individual detection of these markers, suggesting that this combination may represent a potentially novel laboratory method for the clinical diagnosis of CRC. However, the sample size in the current study was relatively small. Further larger scale experiments and other disease cases that need to differentiate with CRC such as chronic enteritis need to confirm the enhanced diagnostic and prognostic values of combined detection of serum B7-H4 and CEA in CRC patients.

## Figures and Tables

**Table 1:** Clinical material of patient and control groups.

Clinical features	Patient group	Control group	Percentage (% of total)
Gender			
Male	41	14	69.49/58.33
Female	18	10	30.51/41.67
Age			
<60	32	14	54.37/58.33
≥60	27	10	45.63/41.67
T stage			
T1	0		0.00
T2	14		23.73
T3	21		35.59
T4	24		40.68
N stage			
N0	6		10.17
N1	11		18.64
N2	42		71.19
M stage			
M0	48		81.36
M1	11		18.64
TNM stage			
I	2		3.39
II	4		6.78
III	42		71.19
IV	11		18.64
Differentiation			
Low	7		11.86
Middle	47		79.66
High	5		8.48
Histology			
Squamous	2		3.39
Mucoid	57		96.61

Note: T means the depth of tumor cell infiltration; N means the level of lymph node metastasis; M means the condition of metastasis.

**Table 2:** The comparison of serum concentrations of B7-H4/OPN/TPS/CEA between CRC patient group and control group.

Group	*n*	B7-H4	OPN	TPS	CEA
CRC	59	95.78 ± 13.42	116.14 ± 56.31	98.77 ± 27.62	77.17 ± 81.09
Control	24	63.20 ± 15.69	78.63 ± 16.43	76.40 ± 15.04	1.95 ± 1.04
*t*		9.539	2.720	3.738	2.332
*P*		0.001	0.032	0.018	0.008

**Table 3:** The association between serum B7-H4, OPN, TPS, and CEA levels and CRC clinicopathological parameters.

Patient characteristics	*n*	B7-H4 (ng/mL)		OPN (ng/mL)		TPS (U/L)		CEA (ng/mL)	
			*p*		*p*		*p*		*p*
Age									
<60	32	97.64 ± 15.64	0.249	130.06 ± 65.98	0.516	96.25 ± 17.59	0.452	70.58 ± 21.50	0.764
≥ 60	27	93.57 ± 10.06		111.60 ± 47.57		101.74 ± 36.28		85.03 ± 13.54	
Gender									
Male	41	94.38 ± 12.24	0.303	139.78 ± 63.91	0.373	100.01 ± 33.14	0.723	97.14 ± 23.01	0.279
Female	18	98.13 ± 15.23		113.68 ± 48.70		96.66 ± 14.75		76.51 ± 43.64	
Tumor masses									
<5 cm	32	89.19 ± 12.56	0.016	110.87 ± 41.61	0.567	91.23 ± 10.39	0.027	63.24 ± 39.87	0.253
≥5 cm	27	102.68 ± 45.29		113.82 ± 57.77		109.19 ± 30.17		92.71 ± 53.42	
Infiltration									
Inside serosal layer	35	86.75 ± 11.31	0.0001	100.47 ± 42.68	0.040	98.53 ± 11.39	0.962	56.94 ± 59.83	0.365
Reached and outside serosal layer	24	100.41 ± 12.10		145.21 ± 60.06		98.89 ± 33.16		92.61 ± 34.21	
Metastasized									
Nonmetastasized	48	83.34 ± 25.23	0.0041	121.50 ± 28.26	0.015	94.47 ± 29.98	0.021	61.00 ± 33.23	0.069
Metastasized	11	116.34 ± 40.82		110.68 ± 57.50		123.01 ± 44.55		95.00 ± 39.82	
Lymph node metastasis									
<4	17	91.945 ± 19.43	0.0052	108.57 ± 50.32	0.353	98.50 ± 29.17	0.443	74.96 ± 26.85	0.106
≥4	42	110.70 ± 16.52		136.99 ± 63.07		105.15 ± 10.50		81.57 ± 19.16	
Differentiation									
Low	7	91.72 ± 9.62	0.135	113.47 ± 44.02	0.203	98.30 ± 16.91	0.573	90.24 ± 17.66	0.109
Middle	47	101.46 ± 11.48		120.43 ± 29.01		104.52 ± 27.24		82.47 ± 23.09	
High	5	100.42 ± 17.34		121.06 ± 31.33		102.84 ± 15.22		85.24 ± 32.6	
Histology									
Squamous	2	98.72 ± 11.56	0.283	122.47 ± 35.16	0.121	103.88 ± 12.46	0.332	85.58 ± 31.50	0.238
Mucoid	57	96.28 ± 15.34		117.29 ± 40.91		95.45 ± 39.67		75.42 ± 33.54	

**Table 4:** The diagnosis value of serum B7-H4/OPN/TPS/CEA in CRC patients.

Test variable	Sensitivity (%)	Specificity (%)	Youden index
B7-H4	88.2	66.2	0.544
OPN	79.8	61.7	0.415
TPS	76.3	67.5	0.438
CEA	59.3	83.3	0.426
Combinational (B7-H4 + OPN)	99.2	55.9	0.511
Combinational (B7-H4 + TPS)	99.0	60.1	0.591
Combinational (B7-H4 + CEA)	98.9	80.4	0.793

**Table 5:** The comparison of serum concentrations of B7-H4/OPN/TPS/CEA between preoperative and postoperative groups in CRC patients.

Group	*n*	B7-H4 (ng/mL)	OPN (ng/mL)	TPS (U/L)	CEA (ng/mL)
Preoperative	59	95.78 ± 13.42	116.14 ± 56.31	98.77 ± 27.62	77.17 ± 81.09
Postoperative	59	55.77 ± 12.28	78.38 ± 35.59	76.48 ± 13.77	30.52 ± 95.30
*t*		6.070	3.620	5.579	2.661
*P*		0.0064	0.041	0.016	0.012

## Data Availability

The data used to support the findings of this study are available from the corresponding author upon request.
